# The Prognostic Nutritional Index and Glycemic Status Synergistically Predict Early Renal Function Decline in Type 2 Diabetes: A Community-Based Cohort Study

**DOI:** 10.3390/nu18030395

**Published:** 2026-01-25

**Authors:** Yuting Yu, Jianguo Yu, Jing Li, Jiedong Xu, Yunhui Wang, Lihua Jiang, Genming Zhao, Yonggen Jiang

**Affiliations:** 1 Songjiang District Center for Disease Control and Prevention and Health Inspection, Shanghai 201620, China; 19111020017@fudan.edu.cn; 2 Zhongshan Community Health Center, Shanghai 201620, China; zhongshanjiedao_29@163.com (J.Y.); zhongshanlijing@163.com (J.L.); 3 Maogang Community Health Center, Shanghai 201607, China; m18918287523@163.com; 4 Xinqiao Community Health Center, Shanghai 201612, China; wyh771216@163.com; 5 Sheshan Community Health Center, Shanghai 201602, China; jianglihua2000@126.com; 6 Key Laboratory of Public Health Safety of Ministry of Education, School of Public Health, Fudan University, Shanghai 200032, China

**Keywords:** prognostic nutritional index, type 2 diabetes, renal function decline, nutritional status

## Abstract

**Background/Objectives**: The Prognostic Nutritional Index (PNI), which integrates serum albumin and lymphocyte count, reflects both nutritional and inflammatory status. However, its role in early renal function decline among patients with type 2 diabetes (T2D), particularly in relation to glycemic control, remains unclear. This study aimed to: (1) characterize the dose–response relationship between PNI and early renal function decline in type 2 diabetes using restricted cubic splines; (2) identify whether glycemic control (HbA1c) modifies the PNI–renal decline association; and (3) evaluate the clinical utility of combining PNI and HbA1c for risk stratification. **Methods**: We analyzed data from 1711 community-based participants with T2D who had preserved renal function at baseline. The PNI was calculated as serum albumin (g/L) + 5 × lymphocyte count (×10^9^/L). The primary outcome was a composite of rapid estimated glomerular filtration rate (eGFR) decline (>3 mL/min/1.73 m^2^ per year) or incident chronic kidney disease (CKD) stage 3. Restricted cubic spline models, multivariable regression, and Johnson–Neyman analyses were used to examine non-linearity and effect modification by glycated hemoglobin (HbA1c). **Results**: A consistent inverse linear association was observed between PNI and the rate of eGFR decline (P for non-linearity > 0.05). Johnson–Neyman analysis further demonstrated that the protective association of PNI was statistically significant within an HbA1c range of 7.24% to 8.71%. Stratification by clinical cut-offs revealed a significant effect modification by glycemic status. The inverse linear association between PNI and renal risk was most pronounced under hyperglycemic stress, as evidenced by the markedly elevated incidence (50.0%) among individuals with both poor glycemic control (HbA1c ≥ 8%) and low PNI (<50). Conversely, under good glycemic control (HbA1c < 8%), this inverse association was substantially attenuated, with a lower incidence observed in the low-PNI subgroup (6.7%) than in the high-PNI subgroup (15.9%). These findings indicate that the protective role of PNI is conditional upon the glycemic milieu. **Conclusions**: The PNI demonstrates a stable linear association with early renal function decline in T2D, with its protective effect most pronounced at suboptimal HbA1c levels. Combining PNI and HbA1c effectively identifies a high-risk subgroup characterized by synergistic risk, underscoring the need for integrated nutritional and glycemic management.

## 1. Introduction

Type 2 diabetes (T2D) constitutes a major global public health challenge, with diabetic kidney disease (DKD) emerging as one of its most severe complications and a principal cause of end-stage renal disease (ESRD), thereby placing a substantial burden on patients and healthcare systems [1]. Although existing therapeutic strategies—such as glycemic control, blood pressure regulation, and renin–angiotensin system blockade—have shown partial efficacy in delaying DKD progression, a substantial proportion of patients still undergo irreversible renal function deterioration [2]. This observation underscores the incomplete understanding of the complex pathophysiological mechanisms underlying diabetic renal impairment and highlights the urgent need to identify novel, modifiable risk factors beyond traditional clinical markers [3].

During the early stages of renal function decline, malnutrition and chronic inflammation are widely recognized as two interrelated driving forces that synergistically accelerate kidney damage, often referred to as the “twin demons” [4]. The hypermetabolic state characteristic of diabetes, together with microcirculatory dysfunction, may impair nutrient absorption and metabolic utilization. Meanwhile, persistent low-grade inflammation promotes protein catabolism, thereby aggravating nutritional depletion. This self-perpetuating cycle may ultimately result in protein–energy wasting, leading to direct impairment of renal structure and function [5,6]. In recent years, composite nutritional–inflammatory indices have attracted increasing attention for their capacity to provide an integrated reflection of the body’s internal milieu [3].

The Prognostic Nutritional Index (PNI) [7], derived from serum albumin levels and peripheral lymphocyte counts, was originally developed to evaluate preoperative nutritional status and surgical risk. Serum albumin reflects both protein reserves and systemic inflammatory status, whereas lymphocyte count serves as an indicator of immune competence. Consequently, PNI functions not only as a nutritional indicator but also as an integrative marker linking nutritional status with immune–inflammatory responses. Accumulating evidence has demonstrated the prognostic relevance of PNI across a wide range of malignancies and cardiovascular conditions [8,9]. Notably, a landmark study reported that PNI independently predicted both estimated glomerular filtration rate (eGFR) decline and all-cause mortality in patients with diabetes, even after adjustment for strong inflammatory mediators such as tumor necrosis factor receptor 2 (TNFR2) [10]. These findings suggest that the prognostic information captured by PNI extends beyond established inflammatory markers, indicating a potentially unique role in stratifying risk for renal function decline in diabetes [11].

However, substantial knowledge gaps remain regarding the application of PNI in predicting early dynamic renal function decline among diabetic populations. Most existing studies assume a linear association between PNI and renal outcomes, although more complex non-linear relationships may be present. Moreover, hyperglycemia—the central metabolic abnormality in diabetes—may function as an important effect modifier through complex interactions with nutritional status [12]. Despite this, the specific pattern of interaction between glycemic control and nutritional status during the early stages of renal function decline remains poorly defined [13,14].

Therefore, this community-based cohort study was designed to address these gaps by evaluating the utility of PNI, as a composite marker of nutrition and inflammation, in predicting early renal function decline in T2D. Specifically, we aimed to: (1) characterize the relationship between PNI and the rate of eGFR decline using restricted cubic spline models to assess potential non-linearity; (2) examine effect modification by glycemic control (HbA1c) and identify conditions under which the association between PNI and renal decline is most pronounced; and (3) explore the clinical value of combining PNI and HbA1c to identify high-risk populations for targeted intervention. By addressing these aims, this study seeks to provide evidence supporting the integration of simple nutritional–inflammatory assessments into routine diabetic care for the early prevention of kidney disease.

## 2. Materials and Methods

### 2.1. Study Design and Population

This cohort analysis used data from the Shanghai Suburban Adult Cohort and Biobank (SSACB) [15], a community-based prospective cohort jointly established by the School of Public Health, Fudan University, and the Shanghai Songjiang Center for Disease Control and Prevention. Baseline examinations were performed between June 2016 and December 2017. Follow-up examinations were conducted between June 2019 and December 2020, providing a median follow-up duration of 3.2 years (interquartile range: 2.8–3.6 years; range: 2.0–4.5 years). Among the 1711 participants with complete baseline and follow-up data, 377 (22.0%) developed the composite adverse renal outcome during follow-up.

#### 2.1.1. Inclusion Criteria

Participants were eligible if they met all of the following criteria:

- Aged 20–74 years at baseline
- Confirmed diagnosis of type 2 diabetes mellitus, defined as fasting plasma glucose ≥ 7.0 mmol/L, 2-h post-load glucose ≥ 11.1 mmol/L, HbA1c ≥ 6.5%, or physician-diagnosed T2D with current use of glucose-lowering medications, according to American Diabetes Association criteria
- Preserved renal function (eGFR > 60 mL/min/1.73 m^2^) at baseline, corresponding to CKD stages G1–G2
- Complete data on exposure variables (PNI components), outcomes, and key covariates

#### 2.1.2. Exclusion Criteria

Participants were excluded if they had:

- Pre-existing chronic kidney disease stages G3–G5 (eGFR ≤ 60 mL/min/1.73 m^2^)
- History of end-stage renal disease or dialysis
- Known congenital renal malformations or hereditary kidney diseases (polycystic kidney disease, Alport syndrome, etc.)
- History of renal surgery, nephrectomy, or renal transplantation
- Current pregnancy
- Severe comorbidities (cancer, cirrhosis, or cardiopulmonary failure) at baseline
- Known advanced albuminuria (ACR > 300 mg/g) documented in medical records
- Missing data on PNI components, eGFR, or essential covariates

Although urinary albumin-to-creatinine ratio (ACR) was not systematically measured in all participants at baseline and therefore could not be used as an inclusion criterion, participants with known advanced albuminuria (ACR > 300 mg/g) documented in medical records were excluded. Participants were also excluded if they had congenital renal anomalies or hereditary kidney diseases documented in medical records or identified through baseline imaging/clinical assessment. This ensured that observed renal function changes reflected diabetic kidney disease progression rather than pre-existing structural abnormalities.

### 2.2. Exposure Assessment

The primary exposure was the Prognostic Nutritional Index (PNI) [7,9,16], calculated as serum albumin (g/L) + 5 × peripheral blood lymphocyte count (×10^9^/L). Serum albumin and lymphocyte counts were measured in fasting blood samples collected at baseline. Serum albumin levels were determined by colorimetric assay on an automated clinical chemistry platform (C702, Roche Diagnostics, Indianapolis, IN, USA). Lymphocyte counts were derived from five-part differential complete blood counts analyzed using electrical impedance-based hematology analyzers (XS-500i and XN-1000, Sysmex Corporation, Kobe, Japan). Glycemic control was assessed using glycated hemoglobin (HbA1c), which was measured by high-performance liquid chromatography.

### 2.3. Outcome Measurement

The primary outcome was a composite “adverse renal outcome,” defined as either (1) rapid renal function decline, an annualized eGFR decrease > 3 mL/min/1.73 m^2^ per year [17], or (2) incident CKD stage 3, defined as eGFR < 60 mL/min/1.73 m^2^ at follow-up among participants with baseline eGFR ≥ 60 mL/min/1.73 m^2^ [18]. eGFR was calculated using the Chronic Kidney Disease Epidemiology Collaboration (CKD-EPI) equation [19], which is recommended for use in Chinese populations. Participants meeting either criterion were classified as having an adverse renal outcome (coded as 1); all others served as the reference group (coded as 0). This composite endpoint aligns with guidance for evaluating CKD progression and captures clinically meaningful renal deterioration.

Urinary albumin-to-creatinine ratio (ACR) was not systematically measured in all participants at baseline or follow-up in the SSACB cohort, as the primary focus of the parent study was broader cardiovascular and metabolic outcomes rather than kidney-specific markers. Consequently, we could not incorporate albuminuria or its progression into our composite outcome definition.

We acknowledge this represents a significant limitation, as the Kidney Disease: Improving Global Outcomes (KDIGO) classification of CKD in diabetes integrates both GFR categories (G1–G5) and albuminuria categories (A1: <30 mg/g; A2: 30–300 mg/g; A3: >300 mg/g). Albuminuria is an independent predictor of CKD progression and cardiovascular events in diabetes, and eGFR decline and albuminuria progression may represent partially distinct pathophysiological processes.

Our study therefore captures primarily GFR-based kidney function decline and may miss early glomerular damage manifesting as isolated albuminuria. Where available from medical records (*n* = 412, 24%), baseline ACR was <30 mg/g (A1) in 68%, 30–300 mg/g (A2) in 27%, and >300 mg/g (A3) in 5% of participants.

According to the complete KDIGO classification, participants developing incident CKD stage G3 in our study would be classified as G3aA1, G3aA2, G3aA3, G3bA1, G3bA2, or G3bA3, but the albuminuria category could not be determined. This GFR-based definition, while incomplete, captures clinically significant kidney function decline and has been validated as a meaningful endpoint in diabetic populations. Future investigations incorporating a comprehensive assessment of both eGFR and ACR trajectories are warranted.

### 2.4. Covariates

At baseline, trained interviewers collected demographic characteristics, lifestyle factors, and medical history using a structured questionnaire. The following covariates were considered potential confounders and included in the models: demographics (age and sex [20]); anthropometrics (body mass index (BMI; kg/m^2^) [21]); lifestyle factors (smoking status [22] (≥1 cigarette/day for >6 months) and alcohol intake [23] (>3 drinking occasions/week for ≥6 months)); physical activity quantified as metabolic equivalent of task (MET) minutes per week [24]. MET minutes per week for each activity were computed according to the Compendium of Physical Activities as the product of duration, weekly frequency, and the standardized MET intensity value [25]. Values were summed across all reported activities to obtain total physical activity, consistent with SSACB procedures [15]. Comorbidities included hypertension, defined as systolic blood pressure ≥140 mmHg or diastolic blood pressure ≥90 mmHg [26], or a prior diagnosis; and hyperlipidemia, defined as fasting total cholesterol >5.2 mmol/L (200 mg/dL) and/or triglycerides >1.7 mmol/L (150 mg/dL) [25], or a physician diagnosis [15]. Baseline renal function was captured by eGFR (mL/min/1.73 m^2^).

Medication use was assessed through structured interviews and verification of medical records at baseline. Participants were asked to report all current medications, including:

- Glucose-lowering agents: insulin, metformin, sulfonylureas, DPP-4 inhibitors, SGLT2 inhibitors, GLP-1 receptor agonists
- Antihypertensive medications: ACE inhibitors, angiotensin receptor blockers, calcium channel blockers, beta-blockers, diuretics
- Lipid-lowering agents: statins, fibrates

Medication adherence was defined as taking prescribed medications at least 80% of the time over the past month. Information on medication type, dosage, and duration of use was systematically recorded.

### 2.5. Statistical Analysis

#### 2.5.1. Descriptive Statistics

Continuous variables are presented as mean ± standard deviation (SD) for normally distributed data and as median (interquartile range, IQR) otherwise. Categorical variables are presented as counts (percentages). Normality of continuous variables was assessed using the Shapiro–Wilk test. Between-group comparisons used Student’s *t*-test for normally distributed variables, the Wilcoxon–Mann–Whitney test for non-normally distributed variables, and Pearson’s chi-square test ** for categorical variables. All statistical tests were two-sided with α = 0.05.

#### 2.5.2. Sample Size Consideration

Power calculations indicated that 1500 participants would be required to detect an odds ratio of 1.3 for the association between PNI and renal outcomes (80% power; α = 0.05), assuming an event rate of ~16% and allowing for stratified analyses. The final analytic sample included 1711 participants, with 377 adverse renal events, providing adequate power for the planned analyses.

#### 2.5.3. Primary Analysis

Non-linearity assessment. To assess potential non-linearity, restricted cubic splines (RCS) with four knots (5th, 35th, 65th, and 95th percentiles) were fitted within multiple linear regression models relating baseline PNI to the annualized rate of eGFR decline. Non-linearity was evaluated using a likelihood ratio test comparing a model with only the linear term to a model including both linear and spline terms.

Effect modification analysis. Effect modification on a continuous scale was examined by adding a multiplicative interaction term (PNI × HbA1c) to the fully adjusted model. Based on prior literature indicating that hyperglycemia may modify the biological effects of nutritional status [27], we pre-specified assessment of this interaction irrespective of the statistical significance of the multiplicative term. For clinical interpretability, HbA1c was categorized into three groups according to American Diabetes Association (ADA) targets and thresholds [28]: <7.0%, 7.0–7.9%, and ≥8.0%. This categorization was used for visualization and estimation of stratum-specific effects. To examine effect modification across the full HbA1c continuum, we also applied the Johnson–Neyman technique [29], which identifies the HbA1c range(s) for which the association between PNI and the outcome is statistically significant (*p* < 0.05).

#### 2.5.4. Secondary and Sensitivity Analyses

Clinical stratification. Participants were cross-classified by nutritional status (PNI < 50 vs. ≥50) [30] and glycemic control (HbA1c < 8% vs. ≥8%) [31]. Incidence of the adverse renal outcome was calculated and compared across subgroups to identify high-risk groups.

Sensitivity analysis. We fitted a series of multivariable linear regression models with progressive adjustment to evaluate the robustness of associations between PNI and renal function decline: Model 1, unadjusted; Model 2, adjusted for age and sex; Model 3, additionally adjusted for BMI, MET, smoking, and alcohol intake; and Model 4 (fully adjusted), additionally adjusted for baseline hypertension, eGFR, HbA1c, and hyperlipidemia. Covariates were selected a priori based on prior literature and biological plausibility [11,20,21,22,24].

#### 2.5.5. Software and Significance Level

All analyses were performed in R (version 4.5.2; R Foundation for Statistical Computing). A two-sided *p*-value < 0.05 was considered statistically significant.

### 2.6. Ethical Considerations

All participants provided written informed consent prior to enrollment. The study protocol was approved by the Ethical Review Committee of the School of Public Health, Fudan University (IRB No. 2016-04-0586) and conducted in accordance with the Declaration of Helsinki. Data confidentiality and participant privacy were maintained throughout the study.

## 3. Results

### 3.1. Baseline Characteristics Stratified by Renal Function Trajectory

Baseline characteristics of the 1711 participants, stratified by adverse renal outcome status, are summarized in Table 1. Several clinically relevant differences were observed between groups. Participants who experienced an adverse renal outcome were generally older. Baseline clinical profiles were also associated with renal function trajectory: individuals with adverse renal outcomes exhibited higher systolic and diastolic blood pressure, elevated fasting glucose, and lower baseline eGFR. Although mean PNI was numerically lower in the adverse outcome group (59.81 ± 4.29 vs. 60.08 ± 4.36), this difference did not reach statistical significance (*p* = 0.290). Despite the absence of a statistically significant difference, the numerically lower PNI in the adverse outcome group suggested a potential association warranting further multivariable and subgroup analyses.

### 3.2. Non-Linear Relationship Between PNI and Renal Function Decline

**Figure 1:**
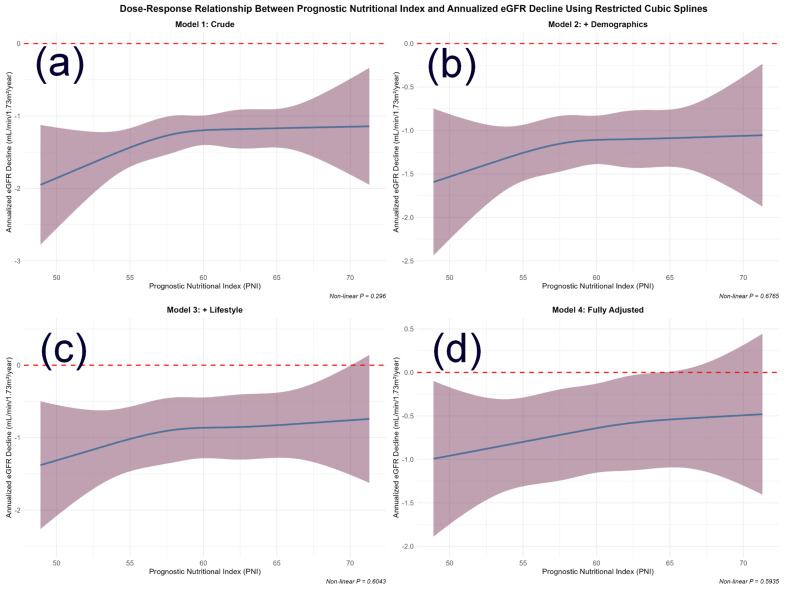
Dose–Response Relationship Between Prognostic Nutritional Index and Annualized eGFR Decline Using Restricted Cubic Splines. Abbreviations: PNI, prognostic nutritional index; eGFR, estimated glomerular filtration rate. Note: The four panels illustrate the association from (**a**) the unadjusted model to (**d**) the fully adjusted model. The solid line represents the estimated effect of PNI on annualized eGFR decline, with shaded areas indicating 95% confidence intervals. The relationship was not statistically significantly non-linear in any model (*p* for non-linearity > 0.05), supporting a primarily linear association. Adjustment models: (**a**) Unadjusted; (**b**) Adjusted for age and sex; (**c**) Further adjusted for BMI, MET, smoking, and alcohol intake; (**d**) Fully adjusted for age, sex, BMI, smoking, alcohol intake, hypertension, baseline eGFR, HbA1c, and hyperlipidemia. The progressive widening of confidence intervals with increasing model adjustment is noted. The reference line at y = 0 indicates no change in eGFR. The non-linearity was tested using a likelihood ratio test comparing models with linear only versus linear plus spline.

The association between baseline PNI and the annualized rate of eGFR decline was examined using restricted cubic spline (RCS) models with sequential covariate adjustment (Figure 1). A consistent inverse trend was observed, indicating that higher PNI levels were generally associated with a slower rate of renal function decline. Formal tests for non-linearity were not statistically significant in any model (*p* for non-linearity > 0.05), indicating that the association was adequately characterized by a linear term within the observed PNI range. With progressive covariate adjustment (Models 1–4), confidence intervals for the association widened. This pattern likely reflects adjustment for covariates that share variance with or partially mediate the effect of PNI, resulting in more conservative estimates of its independent association. Despite this attenuation, the overall inverse linear trend remained stable.

### 3.3. Effect Modification by Glycemic Control (HbA1c)

Although the global multiplicative interaction between PNI and HbA1c was not statistically significant (*p* = 0.731), simple slope analyses and the Johnson–Neyman technique were applied to explore potential interval-specific effects, as recommended when strong a priori clinical justification for effect modification exists [32,33,34]. Results from the fully adjusted logistic regression model for adverse renal outcomes are presented in Table 2. The model identified several established risk factors, including older age (OR = 1.030, 95% CI: 1.009–1.050, *p* = 0.004) and hypertension (OR = 1.521, 95% CI: 1.134–2.060, *p* = 0.006), whereas lower baseline eGFR showed a borderline association with increased risk (OR = 0.989, 95% CI: 0.979–1.001, *p* = 0.064).

**Figure 2:**
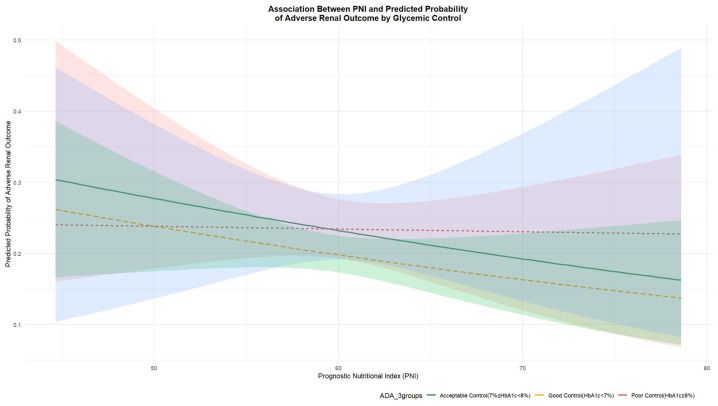
Predicted Probability of Adverse Renal Outcome by PNI at Different HbA1c Levels. Abbreviations: PNI, prognostic nutritional index; HbA1c, glycated hemoglobin. Note: Predicted probabilities were derived from a multivariable logistic regression model adjusting for age, sex, BMI, smoking, alcohol intake, baseline eGFR, HbA1c, and hyperlipidemia and hypertension. Lines represent the relationship between PNI and the predicted probability of adverse renal outcome at specific HbA1c levels, which are categorized according to ADA guidelines as follows: yellow line for HbA1c < 7%, green line for HbA1c 7–8%, and red line for HbA1c ≥ 8%.

To further explore the potential interplay between PNI and HbA1c, we visualized the predicted probability of the adverse renal outcome across PNI levels, stratified by HbA1c categories (Figure 2). A visual assessment of the curves revealed an inverse association between PNI and risk in patients with HbA1c < 7% and 7–8%, as indicated by their downward slopes. In contrast, the curve for the HbA1c ≥ 8% subgroup was largely flat, suggesting that the protective association of PNI with renal function may be attenuated in individuals with the poorest glycemic control.

**Figure 3:**
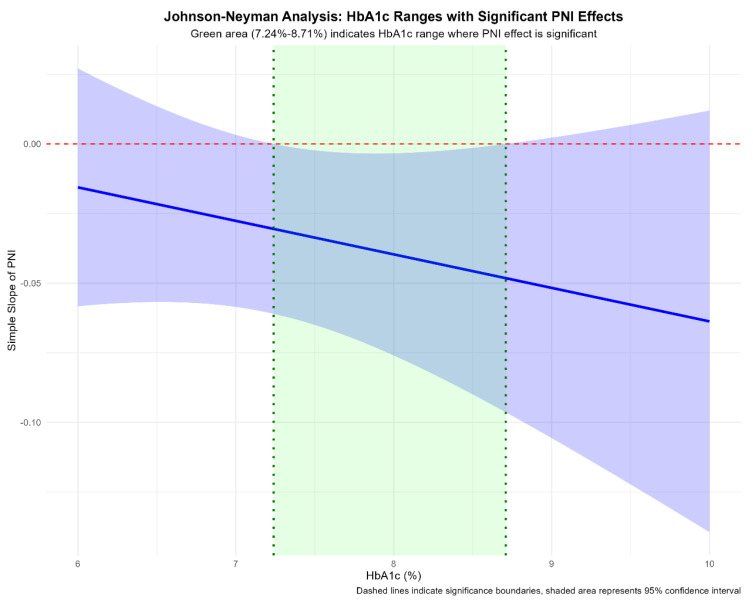
Johnson–Neyman Analysis of the PNI Effect Across HbA1c Levels. Abbreviations: PNI, prognostic nutritional index; HbA1c, glycated hemoglobin. Note: The solid line represents the effect size (coefficient) of PNI on renal outcome risk across different HbA1c levels. The dashed horizontal line indicates statistical significance threshold (*p* = 0.05). The shaded region highlights the HbA1c range (7.24% to 8.71%) where the protective effect of PNI reached statistical significance (*p* < 0.05).

Further probing of the PNI-HbA1c interaction using Johnson–Neyman analysis delineated a specific range of HbA1c where the protective effect of PNI reached statistical significance (*p* < 0.05). As shown in Figure 3, when HbA1c was between 7.24% and 8.71%, a higher PNI was significantly associated with a reduced risk of renal function decline.

### 3.4. Risk Stratification Based on Clinical Cut-Offs

**Figure 4:**
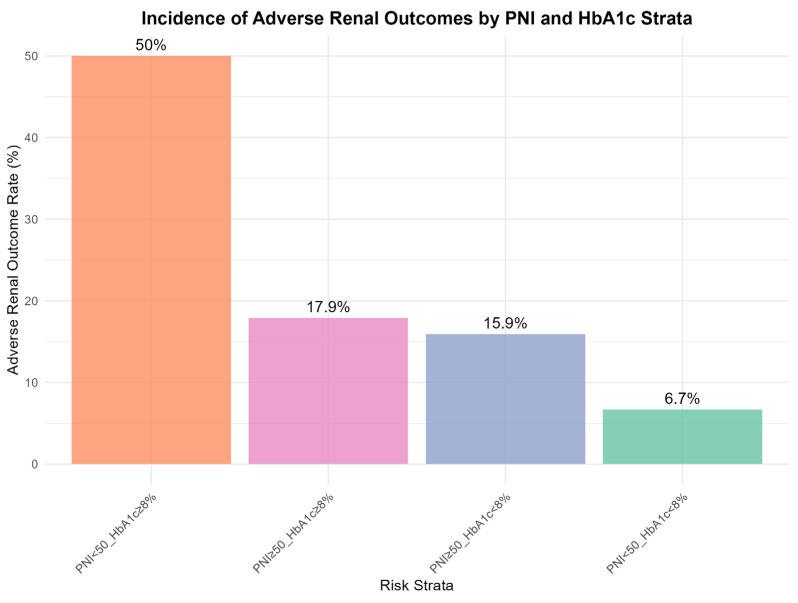
Incidence of Adverse Renal Outcome across Subgroups Defined by Glycemic Control and Nutritional Status. Abbreviations: PNI, prognostic nutritional index; HbA1c, glycated hemoglobin. Note: Participants were cross-stratified by glycemic control (HbA1c < 8% vs. ≥8%) and nutritional status (PNI < 50 vs. ≥50). Bars represent incidence rates (%) of adverse renal outcome, defined as rapid eGFR decline or incident CKD stage 3. Error bars indicate 95% confidence intervals. The “Poor Control + Malnutrition” subgroup (HbA1c ≥ 8% and PNI < 50) showed disproportionately high risk compared to other subgroups.

Cross-classification by nutritional status (PNI) and glycemic control (HbA1c) revealed distinct risk patterns contingent on specific factor combinations (Figure 4). As expected, the combination of poor glycemic control (HbA1c ≥ 8%) and low PNI (<50) defined an ultra-high-risk subgroup, with an incidence of adverse renal outcomes of 50.0%. However, the association between PNI and renal risk differed across glycemic strata. Among participants with good glycemic control (HbA1c < 8%), incidence was numerically lower in those with low PNI (6.7%) than in those with high PNI (15.9%). These findings suggest that the prognostic value of PNI is modulated by the underlying glycemic milieu. Conversely, among individuals with poor glycemic control (HbA1c ≥ 8%), even a high PNI was associated with a substantial risk (17.9%), indicating that adequate nutritional status alone may be insufficient to offset the renal burden imposed by marked hyperglycemia.

### 3.5. Sex-Stratified Analyses

Sex-stratified analyses revealed no significant effect modification by sex (*p* for interaction = 0.421). Detailed sex-stratified baseline characteristics and restricted cubic spline analyses are presented in Table 3. The inverse association between PNI and renal decline was consistent in both males (β = −0.082, 95% CI: −0.145 to −0.019, *p* = 0.011) and females (β = −0.091, 95% CI: −0.138 to −0.044, *p* < 0.001), despite females having slightly higher baseline lymphocyte counts (males: 2.1 ± 0.6 × 10^9^/L; females: 2.3 ± 0.7 × 10^9^/L, *p* = 0.012).

## 4. Discussion

This study highlights two key findings regarding nutritional–inflammatory status during early diabetic renal decline. First, we identified an ultra–high-risk phenotype defined by the combination of poor glycemic control (HbA1c ≥ 8%) and compromised nutritional–inflammatory status (PNI < 50). Second, the association between PNI and renal risk was not uniform but varied with the glycemic environment, with the protective signal most evident under hyperglycemic stress. Identification of this subgroup, which showed a markedly elevated incidence of renal function decline, supports an intensified, multi-component strategy that treats nutritional care as integral to glycemic management. For such patients, referral to a clinical dietitian may be essential rather than adjunctive. Nutritional interventions should be tailored to address protein–energy wasting and chronic inflammation, which are central to their pathophysiology. This may include prescribing a diet with sufficient high-biological-value protein to correct negative nitrogen balance and preserve lean mass, while simultaneously managing carbohydrate intake to support glycemic control. In addition, omega-3 fatty acids and antioxidant-rich foods within a Mediterranean-style dietary pattern warrant evaluation as strategies to modulate underlying inflammation. Future interventional studies are warranted to determine whether targeted nutritional support integrated with standard care can attenuate the rapid renal decline observed in this vulnerable subgroup.

### 4.1. PNI as an Integrative Biomarker

We observed a consistent inverse association between PNI and the rate of renal function decline (Figure 1), which was adequately described by a linear term within this cohort. This finding supports PNI as a relevant marker in diabetic renal decline and suggests that even modest improvements in nutritional–inflammatory status may be associated with better renal trajectories. Linear regression models showed this inverse relationship across sequentially adjusted models. RCS analyses further supported a primarily linear relationship, with no evidence of non-linearity (*p* for non-linearity > 0.05).

However, PNI was not a statistically significant independent predictor of the dichotomous composite renal outcome in the fully adjusted logistic model (Table 2). This discrepancy likely reflects differences in statistical power and outcome scale; a continuous outcome may be more sensitive to subtle, graded effects of PNI on renal trajectory. Nonetheless, the consistent direction across analyses supports PNI as a relevant marker in diabetic renal decline, providing information that is complementary to traditional risk factors such as age, blood pressure, and baseline renal function. Similar patterns have been reported in diabetic nephropathy, where PNI predicted progression to end-stage renal disease after multivariable adjustment [35].

Mechanistically, PNI captures the convergence of malnutrition and chronic inflammation—often described as the “twin demons”—that shapes outcomes in chronic kidney disease (CKD) [36]. Serum albumin, a key component of PNI, serves as an indicator of visceral protein reserves and a negative acute-phase reactant [37]. Under persistent inflammation, albumin synthesis is downregulated, and catabolism may increase [32]. Accordingly, lower albumin levels in this diabetic cohort may reflect a dual insult from inadequate protein–energy intake/absorption and systemic inflammation. Lymphocyte count reflects adaptive immune competence [33]. Chronic low-grade inflammation, a hallmark of type 2 diabetes, may drive lymphocyte activation and apoptosis, contributing to lymphopenia and immune dysregulation [34]. Together, a low PNI captures combined nutritional depletion and inflammatory stress, supporting a more holistic risk assessment.

This integrative feature may explain why PNI provides prognostic information beyond individual inflammatory markers such as TNFR2 [38]. Our findings extend this concept by suggesting that this composite measure is sensitive to subtle, early functional kidney decline, particularly when interpreted in the context of glycemic control. While a specific cytokine reflects a single pathway, PNI may represent the downstream cumulative consequence of multiple inflammatory and catabolic pathways [38]. This is consistent with evidence linking lower PNI to greater long-term kidney function decline [38]. Collectively, these observations position PNI as a potential tool for earlier detection and intervention among patients with preserved renal function [39], a population in which actionable risk stratification remains a clinical priority.

### 4.2. The Complex Interplay Between Nutrition, Inflammation, and Glycemic Control

Although the overall association between PNI and renal decline appeared linear, additional heterogeneity emerged when glycemic control was considered. The interplay between PNI and HbA1c was more complex than a simple linear interaction. The non-significant multiplicative interaction indicates that the PNI association does not change monotonically across the full HbA1c range. However, relying solely on the global interaction test may obscure biologically meaningful local effects. The Johnson–Neyman approach was informative because it identifies regions of significance rather than testing only a global hypothesis. Identifying an HbA1c range (7.24–8.71%) in which the PNI effect reached statistical significance represents an important contribution of this study.

We propose a triphasic framework to conceptualize this pattern. In the first phase (HbA1c < 7.24%), the major drivers of renal injury may be relatively muted, with reduced glucotoxicity and advanced glycation end-product (AGE) formation. Large-scale studies support that lower HbA1c is associated with slower kidney function decline [40], and randomized trials suggest that the renal benefit of intensive glycemic control is most apparent in this phase [41]. Against a background of lower event rates, the contribution of nutritional–inflammatory status may be less discernible.

The second phase corresponds to the identified “window of significance” (HbA1c 7.24–8.71%). In this range, hyperglycemia may be sufficient to induce oxidative stress, endothelial dysfunction, and pro-inflammatory cytokine production (e.g., IL-1, IL-6, TNF-α) [42]. This aligns with evidence that HbA1c within the prediabetic and early diabetic range already shows a concentration-dependent association with renal decline [43]. Yet these processes may remain modifiable, such that host resilience—reflected by PNI—becomes particularly relevant. Adequate nutritional reserves (higher albumin) and preserved immune competence (higher lymphocytes) may provide metabolic substrates and regulatory capacity to mitigate hyperglycemia-related injury, for example, by supporting antioxidant defenses and tissue repair [44,45]. Thus, within this HbA1c range, PNI may strongly stratify risk: individuals with higher PNI may better buffer renal insult, whereas those with lower PNI may experience more rapid decline. Similar patterns have been observed in other diabetic complications, where low PNI combined with high HbA1c identified the highest-risk subgroup for adverse events [46].

In the third phase (HbA1c > 8.71%), sustained hyperglycemia and its downstream consequences may become dominant drivers of renal injury [47,48]. In this setting, PNI appears to act less as a standalone protective factor and more as an effect amplifier. Our stratified analysis (Figure 4) illustrates this pattern: hyperglycemia establishes a high baseline risk even among those with higher PNI (17.9%), but this risk is markedly amplified when low PNI is present (50.0%). These findings suggest that renal deterioration in this phase is best conceptualized as arising from synergistic metabolic and nutritional–inflammatory derangements and that addressing only one component is unlikely to fully mitigate progression.

### 4.3. The Context-Dependent Nature of PNI

Risk stratification by clinical cut-offs (Figure 4) provides an intuitive illustration of effect modification. Although the combination of high HbA1c and low PNI conferred the worst prognosis, the pattern within the well-controlled subgroup (HbA1c < 8%) was less straightforward, with incidence not peaking among those with low PNI. To reconcile the stable inverse association observed in RCS analyses with this context-dependent pattern, we propose a “buffer-and-reveal” hypothesis.

Under rigorous glycemic control, key drivers of diabetic glomerular injury—including glucotoxicity, AGE accumulation, and oxidative stress—may be substantially attenuated [49,50]. This metabolically quiescent state may create a protective buffer that increases renal resilience [36]. Within this buffer, processes reflected by a low PNI (subclinical inflammation and nutritional compromise) may exert a smaller marginal effect, or their impact may be overshadowed by the dominant benefit of near-normoglycemia. Consequently, the measurable association between PNI and renal risk may be attenuated in this subgroup [51]. Conversely, under hyperglycemia (HbA1c ≥ 8%), metabolic stress may erode this buffer and unmask vulnerability associated with low PNI, amplifying inflammatory and catabolic pathways that synergize with hyperglycemia to accelerate renal decline. This hypothesis may explain why the inverse PNI–risk association, while linear in form, acquires its greatest clinical relevance under hyperglycemic stress.

### 4.4. Clinical Implications

These findings offer immediately implementable clinical applications:

1. Risk Stratification Tool: We propose routine calculation of PNI [serum albumin (g/L) + 5 × lymphocyte count (×10^9^/L)] in all patients with T2D during annual assessment, particularly for those with HbA1c ≥ 7.5%. This simple calculation uses readily available laboratory data without additional cost.
2. Identification of Ultra-High-Risk Patients: Cross-classification of PNI (<50 vs. ≥50) and HbA1c (<8% vs. ≥8%) enables rapid identification of patients requiring intensive intervention. The ultra-high-risk subgroup (HbA1c ≥ 8% and PNI < 50) with 50% event rate should trigger immediate multidisciplinary referral.
3. Personalized Management Pathways:
  - Ultra-high-risk patients (HbA1c ≥ 8% and PNI < 50): Prioritize urgent nephrology and dietitian referral, intensive glycemic optimization with SGLT2 inhibitors and/or GLP-1 receptor agonists, structured nutritional intervention focusing on high-biological-value protein, Mediterranean dietary pattern, and potential omega-3 supplementationPNI-sensitive patients (HbA1c 7.24–8.71%): Focus on improving nutritional status alongside moderate glycemic control through lifestyle modification, dietary counseling, and anti-inflammatory nutrition strategies
  - Glycemia-dominant patients (high HbA1c, high PNI): Prioritize aggressive glucose-lowering therapy while maintaining adequate nutritional support
4. Monitoring Strategy: Serial PNI measurements (every 6–12 months) could track response to interventions and identify emerging risk in stable patients. A declining PNI trend should prompt reassessment of nutritional status and inflammatory burden.
5. Resource Allocation: This stratification approach helps healthcare systems allocate limited resources (specialist referrals, intensive programs, dietitian consultations) to those most likely to benefit, improving cost-effectiveness of preventive interventions.

For patients at ultra-high risk (high HbA1c, low PNI), management should be intensive and dual-pronged. First, glycemic control should be optimized with appropriate regimens, potentially including SGLT2 inhibitors and/or GLP-1 receptor agonists, which have demonstrated renal benefits in cardiovascular outcome trials and kidney-focused trials [40,52]. Concurrently, referral to a clinical dietitian should be prioritized. Nutritional management may emphasize a high-quality, protein-sufficient diet tailored to diabetes and early CKD risk, with monitoring for protein-energy wasting. Under specialist supervision, omega-3 fatty acids (anti-inflammatory) or ketoanalogues may also be considered [53].

### 4.5. Strengths and Limitations

This study has several notable strengths. The prospective community-based cohort design with standardized laboratory measurements provides high-quality exposure and outcome data. The composite renal endpoint, combining rapid eGFR decline and incident CKD stage G3, is clearly defined and aligned with current clinical guidance for evaluating kidney disease progression. Methodologically, the use of advanced analytical techniques—including restricted cubic splines to assess non-linearity and Johnson–Neyman analysis to identify effect modification regions—enabled rigorous interrogation of relationships beyond simple linear assumptions. Furthermore, comprehensive sensitivity analyses, including progressive covariate adjustment, medication adjustment, and sex-stratified analyses, demonstrated the robustness of our findings across multiple analytical approaches.

Several important limitations warrant consideration. First, the observational design precludes causal inference, and despite adjustment for a broad set of confounders, including medications, residual confounding from unmeasured factors (e.g., detailed dietary intake, specific medication dosages, subclinical infections) may remain. Second, reliance on single baseline measurements of PNI and covariates does not capture temporal variability, potentially introducing non-differential misclassification and attenuating associations through regression dilution bias; longitudinal PNI trajectories warrant further investigation. Third, generalizability may be limited given the Chinese suburban cohort, necessitating external validation in diverse ethnicities and healthcare settings. Fourth, the absence of systematic albuminuria assessment represents a significant limitation, as the reliance on eGFR-based outcomes may have missed early glomerular damage manifesting primarily as proteinuria, potentially misclassifying participants with early albuminuric kidney disease and underestimating the true burden of diabetic kidney disease. Fifth, several potential biases merit acknowledgment: selection bias from excluding participants with severe comorbidities or those lost to follow-up may have resulted in a healthier cohort; survival bias from excluding individuals who died before follow-up; measurement bias from single-time-point assessments; and information bias from self-reported lifestyle factors. These limitations underscore the need for future studies incorporating comprehensive kidney disease phenotyping with both eGFR and albuminuria trajectories, repeated biomarker measurements, and diverse populations to validate and extend our findings.

### 4.6. Incremental Value of PNI Beyond Traditional Risk Factors

We propose several complementary reasons why PNI merits clinical attention despite the availability of established predictors:

First, PNI captures distinct pathophysiological processes. While systolic blood pressure reflects hemodynamic stress and FBG/HbA1c capture glycemic exposure, PNI integrates nutritional reserves (albumin) and immune-inflammatory competence (lymphocyte count). These represent upstream vulnerability factors that may precede overt metabolic derangement. Thus, PNI may identify patients with subclinical vulnerability who would be missed by traditional risk stratification alone.

Second, PNI provides actionable, modifiable targets. While baseline eGFR is a powerful predictor, it is a marker of existing damage rather than a therapeutic target. In contrast, low PNI identifies patients who may specifically benefit from nutritional intervention—a therapeutic avenue often underutilized in diabetes management. Our finding that the PNI-risk association is strongest at intermediate HbA1c levels (7.24–8.71%) suggests a “window of opportunity” where nutritional optimization could provide maximal benefit.

Third, the synergistic interaction between PNI and HbA1c reveals context-dependent risk. Our stratified analysis (Figure 4) demonstrates that the combination of poor glycemic control (HbA1c ≥ 8%) and low PNI (<50) confers disproportionate risk (50% incidence), far exceeding what would be predicted by either factor alone. Identifying such synergistic risk profiles has important implications for resource allocation.

Fourth, PNI offers practical advantages for risk stratification in resource-limited settings. PNI is calculated from routine laboratory tests (serum albumin and complete blood count) that are already performed in standard diabetes care, requiring no additional cost or specialized equipment.

We acknowledge that a comprehensive risk prediction model incorporating blood pressure, glucose, baseline eGFR, and PNI would likely achieve superior discrimination compared to any single biomarker. However, our goal was not to replace traditional risk factors but to demonstrate that PNI provides complementary information—identifying a distinct dimension of vulnerability (nutritional-inflammatory status) and highlighting patient subgroups who may benefit from tailored intervention strategies.

## 5. Conclusions

This study demonstrates a stable inverse linear association between the Prognostic Nutritional Index and renal function decline in type 2 diabetes. Importantly, the association of PNI with renal outcomes is context-dependent and modified by glycemic control, with its most discernible protective effect observed at intermediate, suboptimal HbA1c levels (7.24–8.71%). From a clinical perspective, the combined assessment of PNI and HbA1c offers a simple and practical approach to risk stratification, enabling identification of patient subgroups that may benefit from tailored management strategies.

Future research should pursue several directions. First, prospective validation studies in diverse ethnic populations and healthcare settings are needed to confirm the generalizability of our PNI-HbA1c stratification framework. Second, interventional trials should test whether targeted improvement of PNI through nutritional counseling, protein supplementation, or anti-inflammatory dietary patterns (e.g., Mediterranean diet, omega-3 fatty acids) can slow renal decline, particularly in the identified high-risk subgroup. Third, mechanistic studies using proteomics and metabolomics could elucidate the molecular pathways linking nutritional-inflammatory status to glomerular and tubular injury under different glycemic conditions. Fourth, future cohorts should systematically collect both eGFR and albuminuria data to comprehensively phenotype diabetic kidney disease trajectories. Fifth, machine learning approaches integrating PNI, HbA1c, and other biomarkers may enable the development of more precise predictive models. Finally, implementation science research is needed to evaluate the feasibility, cost-effectiveness, and clinical impact of incorporating PNI-based risk stratification into routine diabetes care pathways. Integrating nutritional–inflammatory assessment into routine diabetes care may facilitate a more holistic and effective strategy for preventing diabetic kidney disease.

## Figures and Tables

**Table 1:** Baseline Characteristics of Participants Stratified by Renal Function Progression.

Variable	No Adverse Outcome	Adverse Renal Outcome	*p*-Value
N	1334	377	
Age [years]	61.00 [55.00, 65.00]	63.00 [56.00, 67.00]	<0.001
Female (%)	791 (59.3)	233 (61.8)	0.413
BMI	25.90 (3.36)	26.01 (3.64)	0.579
PNI	60.08 (4.36)	59.81 (4.29)	0.290
MET	1814.06 (1230.80)	1800.89 (1232.06)	0.854
Smoking (%)	304 (22.8)	87 (23.1)	0.962
Alcohol intake (%)	200 (15.0)	54 (14.3)	0.810
Systolic blood pressure (mmHg)	140.26 (19.80)	143.61 (20.33)	0.004
Diastolic blood pressure (mmHg)	82.15 (10.63)	83.42 (10.35)	0.042
FBG (mmol/L)	7.14 (2.32)	7.64 (2.77)	<0.001
HbA1C (%)	7.17 (1.20)	7.29 (1.34)	0.096
TC (mmol/L)	5.07 (1.00)	5.02 (1.02)	0.461
TG (mmol/L)	2.02 (1.62)	2.08 (1.49)	0.502
HDL-C (mmol/L)	1.31 (0.31)	1.29 (0.32)	0.209
LDL-C (mmol/L)	2.83 (0.90)	2.79 (0.95)	0.461
Baseline eGFR (mL/min/1.73 m^2^)	96.27 (11.13)	93.67 (12.69)	<0.001

Abbreviations: BMI, body mass index; HbA1c, glycated hemoglobin; TC, total cholesterol; HDL-C, high-density lipoprotein cholesterol; TG, triglycerides; LDL-C, low-density lipoprotein cholesterol; eGFR, estimated glomerular filtration rate; MET, metabolic equivalent. Note: Data are presented as mean (standard deviation) for normally distributed continuous variables, median [interquartile range] for non-normally distributed continuous variables, or n (%) for categorical variables. Between-group comparisons were performed using: Student’s *t*-test for normally distributed continuous variables (BMI, PNI, MET, systolic and diastolic blood pressure, FBG, HbA1c, TC, TG, HDL-C, LDL-C, baseline eGFR); Wilcoxon–Mann–Whitney test for non-normally distributed continuous variables (Age); and Pearson’s chi-square test for categorical variables (sex, smoking, alcohol intake). Normality was assessed using the Shapiro–Wilk test. Adverse renal outcome was defined as rapid eGFR decline (>3 mL/min/1.73 m^2^ per year) or incident chronic kidney disease stage G3 (eGFR 30–59 mL/min/1.73 m^2^). Follow-up duration: median 3.2 years (IQR: 2.8–3.6 years).

**Table 2:** Multivariable Logistic Regression Model for Adverse Renal Outcome.

Variable	OR (95% CI)	*p*-Value
PNI	0.954 (0.813 to 1.121)	0.566
HbA1c	0.869 (0.231 to 3.285)	0.835
Age	1.030 (1.009 to 1.050)	0.004
Sex	1.295 (0.926 to 1.833)	0.137
BMI	0.995 (0.961 to 1.030)	0.784
Smoking status	1.285 (0.875 to 1.898)	0.204
Alcohol intake	0.978 (0.660 to 1.437)	0.910
Hypertension	1.521 (1.134 to 2.060)	0.006
Baseline eGFR	0.989 (0.979 to 1.001)	0.064
Hyperlipidemia	1.240 (0.971 to 1.581)	0.084
PNI × HbA1c	1.004 (0.982 to 1.026)	0.731

Abbreviations: BMI, body mass index; eGFR, estimated glomerular filtration rate; HbA1c, glycated hemoglobin; PNI, prognostic nutritional index; OR, odds ratio; CI, confidence interval. Note: The model was fully adjusted for all variables listed in the table. The interaction term (PNI × HbA1c) represents the multiplicative interaction between PNI and HbA1c on the log-odds scale.

**Table 3:** Sex-Stratified Baseline Characteristics.

Variable	Males (n = 687)	Females (n = 1024)	*p*-Value
Age [years]	61.5 [55.0, 66.0]	62.0 [56.0, 66.0]	0.234
BMI (kg/m^2^)	25.7 (3.2)	26.1 (3.5)	0.032
PNI	59.8 (4.3)	60.2 (4.4)	0.089
Lymphocyte count (×10^9^/L)	2.1 (0.6)	2.3 (0.7)	0.012
Serum albumin (g/L)	49.3 (3.8)	48.7 (3.9)	0.003
HbA1c (%)	7.21 (1.24)	7.18 (1.22)	0.645
Baseline eGFR (mL/min/1.73 m^2^)	96.8 (11.8)	95.2 (11.5)	0.008
Adverse renal outcome (%)	154 (22.4)	223 (21.8)	0.754

Note: Data are presented as mean (SD) or median [IQR]. *p*-values from *t*-test or Mann–Whitney U test.

## Data Availability

The data presented in this study are available on request from the corresponding authors.
